# Quorum Sensing Signals Alter *in vitro* Soil Virus Abundance and Bacterial Community Composition

**DOI:** 10.3389/fmicb.2020.01287

**Published:** 2020-06-10

**Authors:** Xiaolong Liang, Regan E. Wagner, Bingxue Li, Ning Zhang, Mark Radosevich

**Affiliations:** ^1^Department of Biosystems Engineering and Soil Science, The University of Tennessee, Knoxville, Knoxville, TN, United States; ^2^College of Land and Environment, Shenyang Agricultural University, Shenyang, China; ^3^College of Biotechnology, Shenyang Agricultural University, Shenyang, China

**Keywords:** soil, prophage, induction, community, diversity, quorum sensing

## Abstract

Cell-density dependent quorum sensing (QS) is fundamental for many coordinated behaviors among bacteria. Most recently several studies have revealed a role for bacterial QS communication in bacteriophage (phage) reproductive decisions. However, QS based phage-host interactions remain largely unknown, with the mechanistic details revealed for only a few phage-host pairs and a dearth of information available at the microbial community level. Here we report on the specific action of eight different individual QS signals (acyl-homoserine lactones; AHLs varying in acyl-chain length from four to 14 carbon atoms) on prophage induction in soil microbial communities. We show QS autoinducers, triggered prophage induction in soil bacteria and the response was significant enough to alter bacterial community composition *in vitro*. AHL treatment significantly decreased the bacterial diversity (Shannon Index) but did not significantly impact species richness. Exposure to short chain-length AHLs resulted in a decrease in the abundance of different taxa than exposure to higher molecular weight AHLs. Each AHL targeted a different subset of bacterial taxa. Our observations indicate that individual AHLs may trigger prophage induction in different bacterial taxa leading to changes in microbial community structure. The findings also have implications for the role of phage-host interactions in ecologically significant processes such as biogeochemical cycles, and phage mediated transfer of host genes, e.g., photosynthesis and heavy metal/antibiotic resistance.

## Introduction

Bacteriophages may infect bacterial host cells via lytic and lysogenic cycles, both of which have shown ecological significance. For instance, lytic cycles of reproduction can impact population and community dynamics through lysis of host cells effectively re-routing dissolved organic carbon and other nutrients back to the dissolved pool, a process referred to as the “viral shunt” (Danovaro et al., 2008; Sullivan et al., 2017). Lysogenic cycles in which the phage genome is inserted into the host cell genome without killing the host, may promote host fitness and regulate metabolic functions through selective expression of certain phage encoded genes and transcriptional regulators without production of progeny phage particles (Hurwitz and U’Ren, 2016; Howard-Varona et al., 2017; Williamson et al., 2017). Among the temperate phage, the mechanisms that control lysis-lysogeny decisions in natural environments remain unknown.

The “piggyback-the-winner” (PtW) theory of phage-host population dynamics predicts that high microbial cell densities promote lytic to temperate (lysogenic) switching, highlighting the importance of lysogenic reproductive cycles at high host cell abundances (Knowles et al., 2016). Some microscopic counting-based examinations and viral metagenomic analyses provide evidence for PtW theory (Reyes et al., 2010; Knowles et al., 2016). In contrast, the “kill-the-winner” (KtW) theory predicts that lytic infections are more prevalent and suppress the fastest growing hosts during times of high host cell densities, while lysogenic conversions are stimulated at low host cell abundances (Thingstad and Lignell, 1997; Weitz and Dushoff, 2008; Maslov and Sneppen, 2017). The long-standing KtW paradigm has also gained empirical support (Payet and Suttle, 2013; Brum et al., 2016; Liang et al., 2019c). Both PtW and KtW suggest host-cell density may guide the viral reproductive strategies, although the paradigms propose contrasting fashions of host-cell density influences. Thus, cell density-dependent quorum sensing (QS) might have an important role in the lysogeny-lysis switch of temperate phages. Most recently, the molecular communication between viruses and between viruses and bacteria has shed light on the mechanism underpinning the phage lysogeny-lysis decisions in a few phage-host model systems (Erez et al., 2017; Dou et al., 2018; Liang and Radosevich, 2019; Silpe and Bassler, 2019a).

Quorum sensing functions as cell-density dependent communication among bacteria and enables coordinated gene expression following fluctuations in population density (Miller and Bassler, 2001; McCready et al., 2019). QS bacteria produce signaling molecules, such as different types of N-Acyl homoserine lactones (AHLs), with the concentration of released signaling molecules dependent upon bacterial population density (Fuqua and Greenberg, 2002; Jemielita et al., 2018; Wellington and Greenberg, 2019). Thus, QS plays a major role in adaptive survival and collective activity of bacterial communities. In an initial investigation evaluating the potential impact of QS on lysogeny-lysis switching, Ghosh et al. (2009) assessed the prophage induction response to exogenously added AHL mixtures of N- (butyl, heptanoyl, hexanoyl, ß-ketocaproyl, octanoyl, and tetradecanoyl) homoserine lactones and demonstrated that AHLs triggered viral production (i.e., switching from lysogenic to lytic viral reproduction) in soil and groundwater bacteria. The AHL-mediated prophage induction mechanism was demonstrated to be an SOS-independent process by using the single-gene knock-out mutation in the model system of *Escherichia coli* with λ-prophage (Ghosh et al., 2009). Similar studies by Silpe and Bassler (2019a;b) revealed that the lysogeny-lysis switch of a *Vibrio* phage can be induced by the host-produced QS autoinducers, in which the phage lysogeny-lysis decisions directly respond to host QS molecular signals and cell density. It is important to note that microbially produced QS molecules in Silpe and Bassler (2019a;b) reports as well as Ghosh et al. (2009) have the same prophage induction response as exogenously added autoinducers suggesting that the QS-mediated prophage-induction mechanisms are likely operative in natural systems. However, the potential significance of this phenomena at the microbial community level has only been demonstrated by Ghosh et al. (2009).

Communication among phages through phage-encoded arbitrium peptides was first described by Erez et al. (2017), and the following studies (Dou et al., 2018; Wang et al., 2018; Gallego del Sol et al., 2019; Stokar-Avihail et al., 2019) revealed the molecular basis for the production, detection, and consequences of the short signaling peptides on phages lysogeny-lysis decisions. Notably, these reports also showed that phages communicate only with their close relatives using a very specific arbitrium peptide, which suggests that phage communication peptides act in a taxon-specific manner just as bacterial QS signals. Inspired by the above studies, especially the phage-bacterium QS connections, we hypothesized that any single QS signal should only induce prophages within a small subset of closely related host bacteria. Toward that end, we tried to determine the impacts of the addition of individual AHL signaling molecules on prophage-induction and further assess the resulting impact of phage-mediated host cell lysis on bacterial community composition *in vitro* using microbial communities extracted directly from soil.

## Materials and Methods

### Sample Collection and Bacterial Extraction

Soil samples were collected from an agricultural field at the East Tennessee Agricultural Research and Education Center (Latitude = 35.899166; Longitude = –83.961120) on March 5, 2019. Soil samples were collected from one location and thoroughly mixed onsite. The extraction of bacterial cells from soil was performed as described elsewhere (Williamson et al., 2005). For sufficient microbial biomass to complete all the induction assays described below, a larger quantity of soil (300 g) was extracted using cold (stored at 4°C) extraction buffer consisting of 10 g/L potassium citrate, 1.44 g/L Na_2_HPO_4_ ⋅ 7H_2_O, and 0.24 g/L KH_2_PO_4_. The soil to extraction buffer ratio was maintained as described in Williamson et al. (2005), and the mixture was blended in a sterilized blender vessel at the maximum speed for 3 min. The extracted bacteria were concentrated by centrifuging the slurries on a cushion of 60% (w/v) Nycodenz solution (Accurate Chemical & Scientific, Westbury, NY, United States) at 4,000 *g* for 20 min at 4°C. Supernatant including the Nycodenz phase that contains the bacterial cells was transferred to 50 ml centrifuge tubes and was then centrifuged at 5,000 × *g* for 20 min. The bacterial pellets were washed twice using sterile extraction buffer and resuspended in autoclaved 0.2 μm-filtered soil extract (Ghosh et al., 2009) after decanting the supernatant to discard extracellular viruses. The resuspended bacterial extracts were pooled and homogenized by gently shaking. The bacteria and viruses were enumerated after incubation via epifluorescence microscopy direct counting as described below.

### Epifluorescence Microscopy Counting

Epifluorescence microscopy was used for enumeration of bacteria and viruses in the solution (Williamson et al., 2005; Liang et al., 2019b). For removal of extracellular DNA, Deoxyribonuclease I (DNase I, 2.5 units/μl, Thermo Scientific) was used to treat all suspensions. After DNase I treatment, each bacterial solution was filtered through a set of filters, a 0.2-μm-pore-size Isopore membrane filter (Merck Millipore Ltd., Cork, Ireland) on top of a glass microfiber filter (Whatman International Ltd., Maidstone, United Kingdom). For viral enumeration, each DNase-treated solution was filtered through a 0.22-μm Millex syringe filter (Merck Millipore Ltd., Tullagreen, Co., Cork, Ireland) to remove bacteria. Viruses were captured by filtering each viral filtrate through a 0.02-μm-pore-size Whatman Anodisc filter (Whatman International Ltd., Maidstone, United Kingdom). The filtration of bacteria and viruses was performed in a Millipore vacuum manifold (Sigma-Aldrich Corporation, St. Louis, MO, United States) under a pressure of less than 62 kPa. The filters containing bacteria and viruses were stained using SYBR gold in a final 2× concentrate (5,000-fold dilution of the original stock solution). The filters were immediately analyzed using a Nikon Eclipse E600 epifluorescence microscopy equipped with FITC filter set and Retiga EX-i CCD camera (Qimaging, Surrey, BC, Canada). Enumeration of bacteria and viruses were performed using IPlab software (BD Biosciences, Franklin Lakes, NJ, United States). For counting bacteria or viruses in each sample, at least 10 fields were digitally photographed at a magnification of 1,000X and an average of bacterial/viral counts was used for further calculation of their density in the sample. The bacterial and viral abundances in each treatment were derived from triplicate samples.

### *In vitro* Prophage Induction With AHLs as Inducing Agents

The pooled and homogenized bacterial extracts was distributed into 30 aliquots in tubes each containing the respective inducing agents for induction assay or controls (Supplementary Figure S1). Sterile soil extract was used as the medium for induction assays to provide native growth substrates and trace elements to support bacterial metabolism and viral reproduction in induced lysogenic cells.

Eight different AHLs, (N- butyryl-, hexanoyl-, β- ketocaproyl-, heptanoyl-, octanoyl-, 3- oxododecanoyl-, tetradecanoyl-, and 3-oxotetradecanoyl-homoserine lactones, Wellington and Greenberg, 2019) varying in molecular weight and structure (Table 1 and Supplementary Figure S2) and mitomycin C were selected for induction assays. The presence and production of these AHL molecules in soil has been demonstrated by *in situ* characterization of AHL production from both soil bacterial isolations and indigenous soil communities (Burmølle et al., 2005; Huang et al., 2013; Erdönmez et al., 2017). The selected AHLs were designated AHL1 to 8 based on their molecular weight from lowest to highest (acyl C chain length refer to Table 1). Each AHL was dissolved in ethyl acetate acidified with acetic acid (0.1%, vol/vol) as stock solution and applied at a final concentration of 1 μM in the bacterial suspension. The required amount of each AHL compound in stock solution was added into glass tubes, and the tubes were gently shaken for evaporation of the solvents so that the AHL compounds bonded to the tube bottom as a film (Ghosh et al., 2009).

**TABLE 1:** homoserine lactones (AHL1–8) and mitomycin C (MIT), used as prophage-inducing agents in this study.

**Symbol**	**Name**	**Molecular weight**	**Formula**
AHL1	N-Butyryl-DL-homoserine lactone	171	C_8_H_13_NO_3_
AHL2	N-Hexanoyl-DL-homoserine lactone	199	C_10_H_17_NO_3_
AHL3	N-(β-Ketocaproyl)-L-homoserine lactone	213	C_18_H_33_NO_3_
AHL4	N-Heptanoyl-L-homoserine lactone	213	C_12_H_21_NO_3_
AHL5	N-Octanoyl-DL-homoserine lactone	227	C_18_H_31_NO_4_
AHL6	N-(3-Oxododecanoyl)-L-homoserine lactone	297	C_10_H_15_NO_4_
AHL7	N-Tetradecanoyl-DL-homoserine lactone	311	C_16_H_27_NO_4_
AHL8	N-(3-Oxotetradecanoyl)-L-homoserine lactone	325	C_11_H_19_NO_3_
MIT	Mitomycin C	334	C_15_H_18_N_4_O_5_

The pooled bacterial extract was distributed into AHLs- and mitomycin C-coated glass tubes, each containing a 10 ml aliquot of bacterial suspension. Ten ml of the exact same bacterial suspension was also distributed to clean glass tubes lacking any inducing agent to serve as control. Each treatment and control were prepared in triplicate. All suspensions were incubated in the dark for 18 h at room temperature (Ghosh et al., 2009). The viruses and bacteria in the suspensions were enumerated using epifluorescence microscopy to determine the induction response due to each inducing agent. Viral and bacterial abundance in each sample was estimated by epifluorescence microscopy enumeration as previously described (Williamson et al., 2007; Liang et al., 2019b).

### Bacterial 16S rRNA Genes Sequencing and Statistical Analysis

After 18 h incubation, 1 ml of each cell suspension from the induction assays was transferred to a new sterile centrifuge tube and treated with DNase I for 20 min. The reaction was terminated by addition of EDTA prior to centrifugation at 5,000 × *g* for 20 min at 4°C. The bacterial pellets were washed twice with sterilized extraction buffer to remove all lysed bacterial cells and any residual undigested free DNA. The genomic DNA of un-lysed bacterial cells that survived prophage induction from each sample was extracted using PowerLyser PowerSoil DNA isolation kit (Qiagen, Hilden, Germany) and quantified with using a Nanodrop one spectrophotometer (Thermo Scientific). The DNA samples were sent to the Genomics Core Laboratory at University of Tennessee (Knoxville, TN, United States) for sequencing. The V3-V4 region of 16S rRNA genes were amplified using PCR primer set (341F_CCTACGGGNGGCWGCAG, and 785R_GACTACHVGGGTATCTAATCC) for library construction, and finally sequenced via 300PE (paired-end) on the Illumina MiSeq platform (Illumina, United States) by using the manufacturers’ protocol.

The obtained sequence data was demultiplexed depending on the barcodes. The raw 16S rRNA gene sequence data with all sequence reads was processed using the MOTHUR v.1.40.0 pipeline according to the MiSeq SOP (Kozich et al., 2013). In general, two sets of sequence reads (forward and reverse) for each sample were merged into contigs in which process the pairs of sequences were aligned with quality score of each base calculated. The total sequence reads from each treatment ranged from 57,608 to 213,551. To reduce sequencing and PCR errors, the resulting 3,821,672 sequences were trimmed of sequences containing ambiguous bases and long polymers. As many of the quality-filtered sequences were identical to each other, the unique sequences were identified and sorted with abundance. Sequences were classified by alignment to the customized SILVA reference (Quast et al., 2013), which pre-clustered sequences by 99% similarity. Following reference alignment, the removal of chimeric sequences was performed by VSEARCH algorithm. The divided sequences classified into OTUs (operational taxonomic unit) at 97% nucleotide identity by using the Bayesian classifier, which resulted in a total of 56,996 OTUs. Statistical analyses of the processed sequence data were performed using software R version 3.6.1 packages phyloseq (McMurdie and Holmes, 2014), vegan (version 2.5-2, Oksanen et al., 2016), ggplot2 (Wickham, 2016), and DESeq2 (Love et al., 2014). Bacterial taxonomic composition and alpha-diversity were calculated, and beta-diversity was also assessed based on Bray-Curtis dissimilarity matrix. The quantification and statistical inference of systematic differences of the bacterial taxonomic composition between each induction assay and the control samples were performed using the package DESeq2 (Love et al., 2014). Variance mean was estimated over all treatments (each was based on three replicate samples and treated independently and identically). The files of raw sequences were archived at the National Center for Biotechnology Information Databases (Sequence Read Archive) and can be obtained under accession number SRR10238150.

## Results

### Prophage Induction

Bacteria were extracted, purified and concentrated from the agricultural soil in a way that eliminated most extracellular viruses as described above for bacterial extraction. In this way, the viral background counts were reduced from 6.3 × 10^8^ to 5.3 × 10^5^ ml^–1^ in the pooled bacterial suspensions. The resulting bacterial suspensions were pooled to create one homogeneous mixture of native soil bacteria for use in prophage induction assays. The pooled cell concentrates (before the 18 h incubation assay period) had a bacterial abundance of 1.28 × 10^8^ cells ml^–1^, and the density of viruses was 5.33 × 10^5^ particles ml^–1^. After the 18 h induction assays, the induced cell suspensions were compared directly to the uninduced control suspensions for variations in viral and cell abundance and bacterial community composition.

**FIGURE 1:**
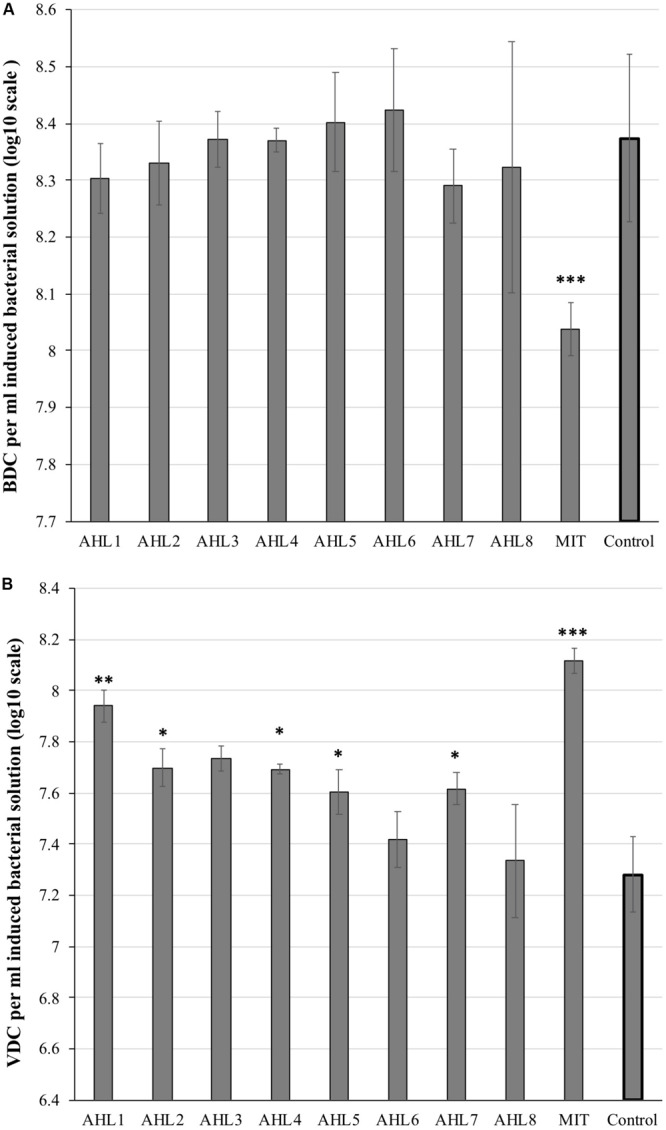
Microscopic direct counts of bacteria **(A)** and viruses **(B)** in the induced and control cell suspensions after prophage induction. Inducing agents included mitomycin C (MIT) and N-(Butyryl, Hexanoyl, β-Ketocaproyl, Heptanoyl, Octanoyl, 3-Oxododecanoyl, Tetradecanoyl, and 3-Oxotetradecanoyl) homoserine lactones (represented as AHL1–8). Control indicates unamended control cell suspensions without AHLs or Mitomycin C treatment. Each bar represents the mean of triplicate assays (*n* = 3), and the error bars show one standard deviation. Statistical significance between the treatment and the control group is indicated by ^∗^*p* < 0.05, ^∗∗^*p* < 0.01, and ^∗∗∗^*p* < 0.001.

Viral and bacterial abundance was quantified to assess the prophage induction response of host cells. The mitomycin C-treated suspensions had significantly lower bacterial abundance and notably higher viral abundance than the control suspensions (*P* < 0.01, *t*-test, Figure 1). No significant decrease in bacterial abundance was observed in the eight AHL-treated suspensions compared to the control samples. The viral abundance in AHL1-, AHL2-, AHL3-, AHL4-, AHL5-, or AHL7-treated suspensions was significantly higher compared with that in the control suspensions (*P* < 0.05, *t*-test, Figure 1B).

### Bacterial Community Diversity and Composition

**FIGURE 2:**
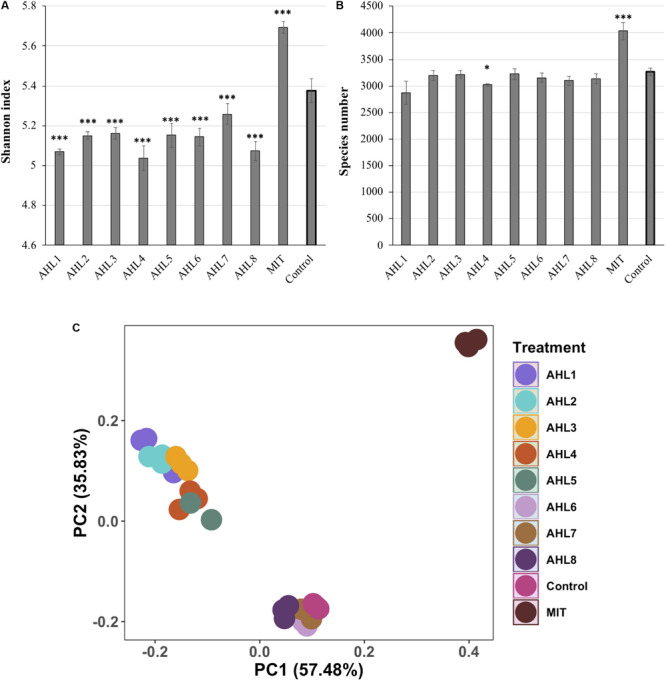
Alpha- and beta- diversity of *in vitro* bacterial cell suspensions. **(A)** Shannon index. **(B)** Bacterial species richness. Data shown as the mean of triplicate cell suspensions assays, and the error bars show one standard deviation. **(C)** Principal component analysis (PCA) of bacterial community composition. Samples are color-coded based on AHL or mitomycin C (MIT) treatment and the control. AHL1–8 stands for induction using quorum sensing signal molecules of N-(Butyryl, Hexanoyl, β-Ketocaproyl, Heptanoyl, Octanoyl, 3-Oxododecanoyl, Tetradecanoyl, or 3-Oxotetradecanoyl) homoserine lactones, respectively. Statistical significance between the treatment and the control group is indicated by ^∗^*p* < 0.05 and ^∗∗∗^*p* < 0.001.

To evaluate the impacts of prophage induction at the community level, the bacterial species richness and alpha diversity were estimated based on species number and Shannon index, respectively as determined by analysis of 16S rRNA gene sequencing of un-lysed cells remaining following the induction assays. The results of 16S rRNA gene sequencing of each treatment were based on three replicate assays, which were treated independently and identically. The bacterial community in mitomycin C-treated suspensions had significantly higher species richness and diversity than that in the control suspensions (*P* < 0.01, *t*-test, Figure 2). In contrast, the bacterial species diversity in AHLs-treated suspensions was lower than that in the control suspensions (*P* < 0.05, *t*-test, Figure 2A). Similar results were obtained for inverse Simpson index (data not shown). There was no statistical difference in bacterial species richness between each AHL treated cell suspension and the control suspensions (Figure 2B).

Principal component analysis (PCA) was used to visualize the dissimilarities of bacterial community structure between suspensions after prophage induction and showed that the communities were separated according to the specific AHL signal compound added to the cell suspensions (Figure 2C). The bacterial community structure in the induction assays using AHL1–5 were notably different from those in the uninduced controls. Some dissimilarity was also observed in bacterial community structure among samples treated with different AHLs. For example, communities resulting from treatment with AHL1-5 were distinctly different from communities treated with AHL6–8 (Figure 2C). mitomycin C-treated suspensions were clearly separated from controls and AHL-treated suspensions, and the first two principal coordinates explained 93.3% of the variation of bacterial community structure (Figure 2C).

### Bacterial Taxonomic Profiles Following Prophage Induction

**FIGURE 3:**
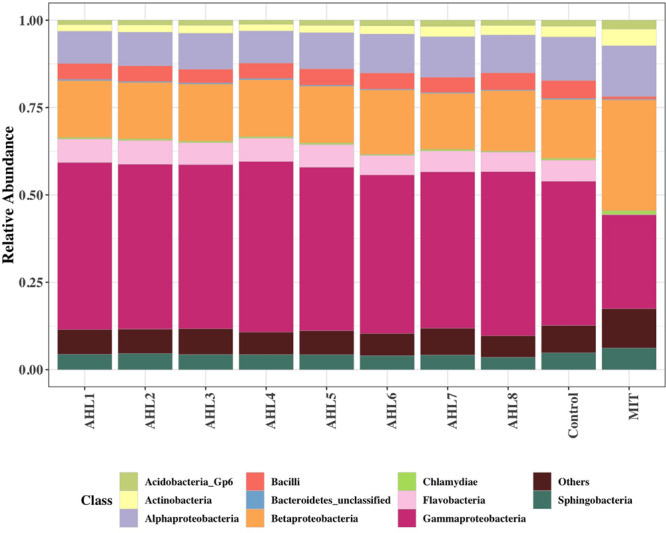
Class-level community composition showing the relative abundances of dominant bacterial classes across all treated samples and the control group. Treatments include induction assays with either N-(Butyryl, Hexanoyl, β-Ketocaproyl, Heptanoyl, Octanoyl, 3-Oxododecanoyl, Tetradecanoyl, and 3-Oxotetradecanoyl) homoserine lactones (represented with AHL1–8, respectively) or mitomycin C (MIT).

**FIGURE 4:**
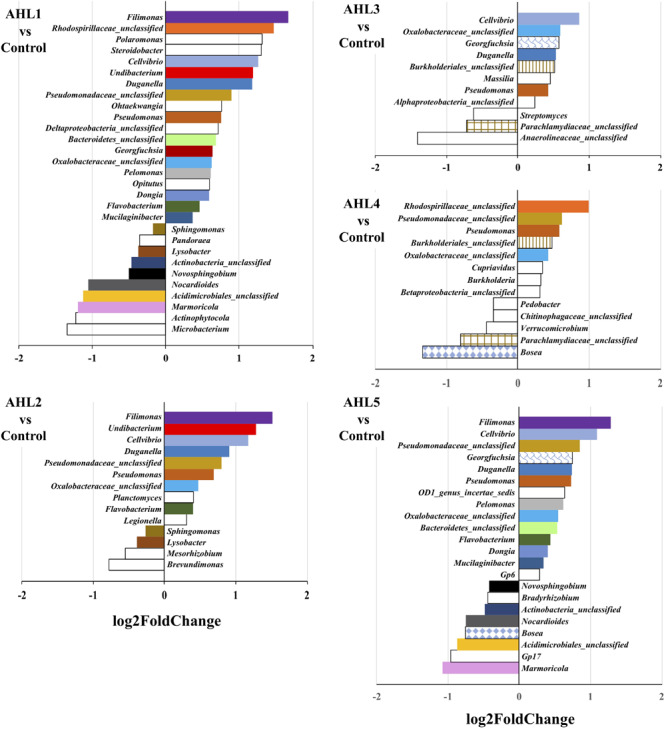
Genus-level differences of the bacterial community composition between each induction assay of N-(Butyryl, Hexanoyl, β-Ketocaproyl, Heptanoyl, Octanoyl, 3-Oxododecanoyl, Tetradecanoyl, and 3-Oxotetradecanoyl) homoserine lactones (indicated by AHL1–8, respectively) and the control suspensions. Only statistically significant differences (*P* < 0.01) were shown. The direction of bars represents decreases (left) or increases (right) in relative abundance of the specific bacterial taxonomic group after the induction assays relative to the unamended controls. Differential analysis of bacterial taxonomic abundances between the treatment and control group was performed using the DESeq2 package for R (Love et al., 2014). Variance mean was estimated over all treatments (each was based on three replicate assays and treated independently and identically). The color of the bar represents taxonomic group shared by two or more different treatments, and the bars without fill represent the taxonomic groups with significant differences compared with the control only detected in the current treatment.

To further examine the differences in bacterial community structure resulting from AHL-mediated prophage induction, we compared the abundance of each bacterial taxonomic group in every induction treatment to that in the control samples at both class and genus level. A stacked bar chart was constructed to show the community composition of the dominant bacterial classes across all groups, and notable differences were revealed between each treatment and the control group (Figure 3). The AHL1 treatment led to a decreased relative abundance of the greatest number of taxa including: Actinobacteria, Alpha-Proteobacteria and Acidobacteria Gp6 at the class level (*P* < 0.01; Figure 3 and Supplementary Figure S3) and *Microbacterium*, *Actinophytocola*, *Mamoricola*, *Nocardioides*, *Novosphingobium*, *Lysobacter*, *Pandoraea*, and *Sphingomonas* at the genus level (*P* < 0.01; Figure 4). AHL2 treatment led to a decreased abundance of two bacterial classes, Actinobacteria and Alpha-Proteobacteria, and genera, *Brevundimonas*, *Mesorhizobium*, *Lysobacter*, and *Sphingomonas* (*P* < 0.01; Figure 4). AHL5 treatment caused a decreased abundance of the second greatest number of bacterial taxa including Acidobacteria Gp6, Gp17 and Actinobacteria at the class level (*P* < 0.01; Figure 3 and Supplementary Figure S3) and *Mamoricola*, *Gp17*, *Bosea*, *Nocardioides*, *Bradyrhizobium*, *Novosphingobium*, and *Acidobacteria Gp6* at the genus level (*P* < 0.01; Figure 4). Treatment with AHL3 and 4 resulted in a decreased abundance of only one class, Chlamydiae. However, AHL3 and 4 had distinct induction effects at the genus level where the abundance of *Streptomyces*, and unclassified *Anaerolinaceae* and *Parachlamydiaceae* declined with AHL3 treatment and *Bosea*, *Verrucomicrobium*, and *Pedobacter* in AHL4 exposure (*P* < 0.01). The AHLs discussed above also resulted in an increased abundance of some bacterial taxa, e.g., classes of Gamma-Proteobacteria, Bacilli and Flavobacteria (*P* < 0.01). It is interesting to note that an increased abundance of *Pseudomonas* was observed in the treatment with AHL1–5 that had significant shifts in the density of specific bacterial groups. The bacterial genera having increased relative abundance after treatment with some AHLs also included *Filimonas*, *Cellvibrio*, *Duganella*, *Pelomonas*, and *Flavobacterium* and certain other unclassified genera (*P* < 0.01). The bacterial taxonomic profiles in the treatment of AHL6, 7, and 8 had no statistical difference compared with the control cell suspensions.

**FIGURE 5:**
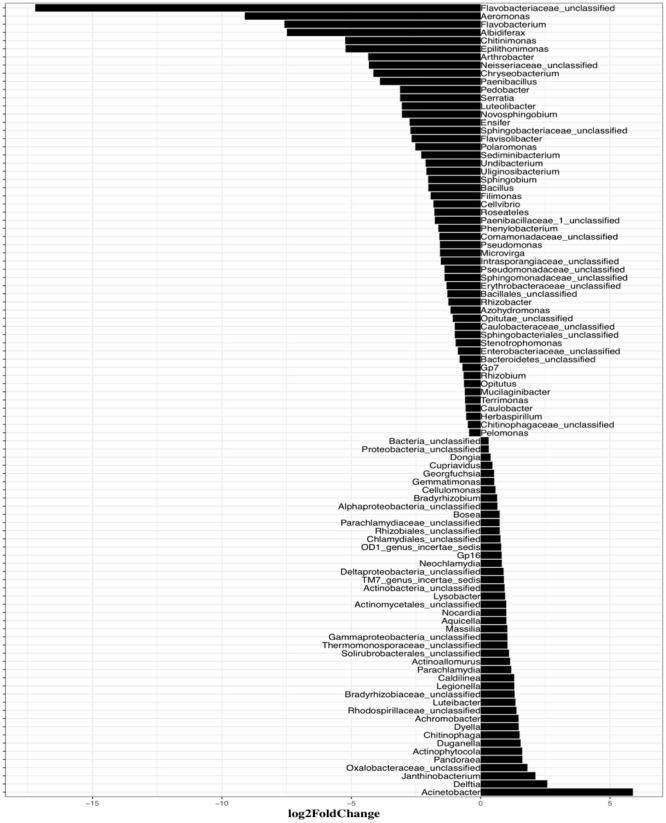
Systematic differences of the bacterial taxonomic composition between induction assay of mitomycin C and the control samples at Genus levels. Only statistically significant differences (*P* < 0.01) are shown. The direction of bars represents decreases **(left)** or increases **(right)** in relative abundance of the specific bacterial taxonomic groups after the induction assays.

Mitomycin C treatment stimulated much broader impacts on the taxonomic composition of the bacterial community than the AHLs suggesting that the prophage induction response brought about by exposure to mitomycin C was less specific than any of the AHLs used as inducing agents. This is consistent with the more general prophage induction response likely brought about by activation of DNA-repair systems associated with mitomycin C-mediated prophage induction. As mitomycin C is an antibiotic, it likely also directly inhibits bacterial growth and causes more cell death leading to the more extensive apparent impacts on the bacterial community composition compared with AHLs treatment. The abundance of six bacterial classes, including Flavobacteria, Bacilli, Gamma-Proteobacteria, Acidobacteria Gp7, Opitutae and Verrucomicrobiae, declined in mitomycin C treated compared to the control suspensions (*P* < 0.01; Figure 5). Up to 53 genus-level bacterial groups, such as *Aeromonas*, *Flavobacterium*, *Albidiferax*, *Chitinimonas*, *Arthrobacter*, *Chryseobacterium* and *Paenibacillus*, had decreased relative abundance after mitomycin C treatment compared with the control suspensions (*P* < 0.01; Supplementary Figure S4). *Cellvibrio*, *Filimonas*, *Pelomonas, Flavobacterium*, and *Pseudomonas*, the bacterial genera that had increased relative abundance after the treatment of some AHLs, had decreased relative abundance in the mitomycin C treatment (*P* < 0.01). The common bacterial genera having decreased relative abundance (comparison between the treatment and control group) and shared by mitomycin C and some AHL treatments included *Novosphingobium* and *Pedobacter*. Mitomycin C exposure also resulted in a slightly increased proportion of 44 bacterial genera (*P* < 0.01; Supplementary Figure S4).

## Discussion

This study revealed the lysogeny-lysis switch of some temperate phages is responsive to QS autoinducers and the phenomenon may be more widespread than the handful of well-characterized phage-host systems reported. The molecular basis of a host QS autoinducer controlling a phage lysogeny-lysis decision has been recently characterized (Silpe and Bassler, 2019a). Subsequent studies reported phage responses to other types of host autoinducers (Laganenka et al., 2019; Silpe and Bassler, 2019b). Bacteria communicate with their close relatives using specific QS signals, thus phage-bacterium QS connections may also be molecular structure dependent (Liang and Radosevich, 2019). Building on this idea, we hypothesized that any single QS signal should only induce prophages within a small subset of closely related host bacteria. In this study, eight AHLs of varying molecular weight and structure were selected for evaluation of phage responses of lysogeny-lysis switching and its significance in structuring the exposed well mixed bacterial communities extracted and purified from a single soil source *in vitro*.

Significant differences in viral abundance were observed in the treated microbial cell suspensions exposed to AHL1, 2, 3, 4, 5, and 7 compared with the control suspensions. The increase of viral abundance in AHL-treated suspensions was consistent with a burst of viral production from prophage induction, especially in a relatively few host taxa, and was in agreement with our hypothesis. Other recent studies also reported prophage induction by QS molecules in bacterial hosts, such as *Enterococcus faecalis* (Rossmann et al., 2015) and *E. coli* (Laganenka et al., 2019), leading to viral production and bacterial lysis. Interestingly, Oh et al. (2019) discovered that though different prophages were induced by environmental cues they achieved differential induction responses indicating a unique governing system for each prophage. A significant decrease in total cell abundance was typically not observed upon exposure to AHLs. This too is consistent with the hypothesis if each AHL triggered prophage induction in a relatively narrow range of perhaps less numerically abundant taxa. In order to test this hypothesis in future studies, investigating bacterial community responses under addition of cocktails of multiple AHLs to the same bacterial suspensions should be considered. Additionally, meta-genomic and meta-transcriptomic analyses during the induction assay could reveal specific prophage-host responses to AHL-exposure. Two other possibilities may also be consistent with the observed results of the treatment-driven changes in viral and bacterial abundances. One possibility is that some of the increased abundance of fluorescence particles from AHL-treated cell suspensions may have been DNA-carrying membrane vesicles secreted/released by AHL-treatment (Toyofuku et al., 2017). Previous reports have indicated that membrane vesicles play important roles in QS and can increase epifluorescence-based viral counts (Li et al., 2016; Biller et al., 2017). The other possibility may be that the produced phages during treatment were released without host lysis (Rakonjac et al., 1999; Xue et al., 2012; Loh et al., 2019). Therefore, future work is needed to elucidate the effects of QS on viral and bacterial populations and advance our understanding of this important phenomenon which may have important ecological consequences in nature. Other experimental approaches that will be useful in confirming the findings reported here might include qPCR quantification of bacterial 16S rRNA gene abundance and phage marker genes, transmission electron microscopy enumeration and morphological description of viral particles.

Even a small collection of lysogenic bacterial taxa triggered to enter the lytic cycle could result in changes in taxonomic composition. We inspected the bacterial community diversity based on species richness and Shannon index. Significantly lower Shannon indices but no significant changes in species richness were observed after AHL treatment. These results suggest that AHL treatment decreased species evenness potentially by suppressing a subset of bacterial species and resulting in increased relative abundance of some other species. Further analysis of Pielou’s evenness across the samples showed that the species evenness was significantly lowered in AHL1 (*P* < 0.05), AHL3 (*P* < 0.05), AHL5 (*P* < 0.01), and AHL7 (*P* < 0.01) compared to the control suspensions which further supported the proposed mechanism of AHL treatment influencing the bacterial community structure. In contrast, mitomycin C treatment, as a broad-spectrum inducing agent, also possessing broad toxicity, commonly used for prophage induction (Williamson et al., 2007; Knowles et al., 2017; Liang et al., 2020), prompted an increase in both species richness and diversity relative to untreated controls. Mitomycin C treatment brought about a significant increase in viral abundance (*P* < 0.001) and notable decrease in bacterial abundance (*P* < 0.01) through a wide range of viral lysis, thus contributing to higher richness and evenness in bacterial taxonomic profiles. The measurement of Pielou’s evenness in these suspensions demonstrated that mitomycin C treatment significantly increased the species evenness compared to the control group (*P* < 0.01).

Since viral production has been shown to be correlated to nutrient cycling and bacterial metabolism, the prophage induction can also contribute to resource redistribution and bacterial community composition (Liang et al., 2019c, b; Oh et al., 2019). To further examine the putative effects of AHL-dependent prophage induction on bacterial community structure, we determined changes in relative abundance (expressed as log2fold changes relative to control cell suspensions) of the affected taxa. Up to 10 bacterial genera decreased in relative abundance after any single AHL treatment, and a total of 23 different genera for all AHL treatments combined. Decreased relative abundance of *Lysobacter*, *Novosphingobium*, *Sphingomonas*, *Bosea*, and *Nocardioides* was observed in at least two AHL treatments. The significant decrease in density of the AHL-targeted bacterial genera, e.g., *Lysobacter*, *Novosphingobium*, and *Sphingomonas*, suggests AHL-directed transition of prophages from lysogeny to lysis in these bacteria. While AHLs caused reduced relative abundances of some bacterial taxonomic groups, AHL treatment also resulted in an increased relative abundance of some bacterial taxa, e.g., classes of Gamma-Proteobacteria, Bacilli and Flavobacteria although the positive affect on some taxa may be indirectly attributed to prophage induction and lysis of other susceptible taxa and/or some growth of the non-susceptible groups. However, if the observed increases in relative abundance were strictly due to growth of certain taxa on the extractable soil dissolved organic carbon (DOC) that was in the induction assays then the patterns would have been similar in all the treatments instead of the differing patterns that were observed. Interestingly, the relative abundance of *Nocardioides* and *Bacilli*, gram-positive genera, varied significantly in the presence of AHL, a QS autoinducer in gram-negative bacteria which may be hard to explain. However, previous studies have shown that many of *Nocardioides* and *Bacilli* members can degrade AHLs leading to quorum quenching (Dong et al., 2002; Yoon et al., 2006; Hong et al., 2012; Vinoj et al., 2014; Kusada et al., 2019) thus likely having undetermined important associations with AHL-producing bacterial groups. Microbial interactions within the community are also an important factor to be considered for variations in community composition and diversity (Tian et al., 2018). However, the explanations of abundance variations of *Nocardioides* and *Bacilli* cannot be demonstrated by the current results in this research.

Quorum sensing has been demonstrated to be widespread among bacteria (Polkade et al., 2016; Liao et al., 2018; Ling et al., 2019), however, AHLs have not been shown as QS signals for many of the AHL-impacted bacterial groups in the present study, such as *Nocardioides*, *Streptomyces*, *Pedobacter*, and *Verrucomicrobium*. While we consider the AHL-mediated prophage induction as the main driving force of differences in the bacterial community composition we observed, other factors should also be considered. For example, QS autoinducers can regulate bacterial collective behaviors such as virulence and biofilm formation and influence inter- and intra-population interactions within the community which thus may influence bacterial community structure (Kim et al., 2016). There are also reports showing that AHLs can be utilized by some bacteria for growth (Huang et al., 2003). So, the selective impact of AHLs themselves needs to be further considered.

Mitomycin C treatment resulted in decreased abundance of 53 bacterial genera. Though the observed decreases may have resulted from virus-mediated host cell lysis upon prophage induction or simply from direct toxicity of the mitomycin C, these broad-spectrum changes clearly contributed to the observed increases in evenness of bacterial species profiles and thus increases in the community diversity. Increased proportion of specific bacterial groups observed in AHL or mitomycin C treatment might be derived from competitive release (Loudon et al., 2014). Growth of some rare bacterial species might also be promoted which contributed to the increased species richness in mitomycin C treated suspensions. Susceptible bacterial species were lysed by chemical induction of prophages allowing the remaining competitors to utilize the resources more fully, and the remaining members of the bacterial community may also have access to the nutrients released through the viral shunt (Danovaro et al., 2008; Kuzyakov and Mason-Jones, 2018).

Lysogeny has been demonstrated to be widespread and a common viral life strategy in nature and shown to have links with the dynamics of the nutrient regime and host density (Howard-Varona et al., 2017). Chemical induction assays, like mitomycin C, have been adopted to assess lysogeny among viral and bacterial communities (Ghosh et al., 2009; Knowles et al., 2017; Kronheim et al., 2018). Though the phage-bacterium connections through QS was recently discovered, the QS based prophage-induction are largely unknown except for a few phage-host pairs none of which were derived from soil, with little known about the influence of prophage induction on microbial community dynamics. In this study, we focused on the inducing effects of eight AHLs among different well-known bacterial autoinducers on microbial community structure. Our findings revealed that a broad range of bacterial taxonomic groups were putatively susceptible to prophage induction by these host autoinducers (i.e., AHLs), and we also demonstrated how transitions from lysogeny to lysis of temperate phages responsive to different host autoinducers can have pivotal roles in influencing bacterial community structure. This research provides theoretical and methodological foundation for future study of phage-bacterium communication and the lysogeny-lysis switch of soil viral communities. For future study, the results reported here should be further investigated by including analysis of lysed bacteria as template for 16S rRNA sequence analyses. In addition, metagenomic and metatranscriptomic analyses before during and after induction assays may reveal more specific and direct evidence supporting QS-controlled lysogeny-lysis switching and the hypothesis addressed in this study may resolve more statistically robust relationships and provide unique high-resolution views of virosphere responses to host autoinducers.

## Data Availability Statement

The datasets generated for this study can be found in the National Center for Biotechnology Information Databases and can be obtained under accession number SRP224599.

## Author Contributions

XL and MR conceived and designed the study. XL, RW, BL, and NZ collected and processed the field samples and conducted the experiments. XL completed sequencing and bioinformatics and worked with MR on data analyses. XL composed the manuscript. All authors contributed to the revisions of the manuscript.

## Conflict of Interest

The authors declare that the research was conducted in the absence of any commercial or financial relationships that could be construed as a potential conflict of interest.

## References

[B1] Biller S. J. McDaniel L. D. Breitbart M. Rogers E. Paul J. H. Chisholm S. W. (2017). Membrane vesicles in sea water: heterogeneous DNA content and implications for viral abundance estimates. *ISME J.* 11 394–404. 10.1038/ismej.2016.134 27824343 PMC5270575

[B2] Brum J. R. Hurwitz B. L. Schofield O. Ducklow H. W. Sullivan M. B. (2016). Seasonal time bombs: dominant temperate viruses affect Southern Ocean microbial dynamics. *ISME J.* 10 437–449. 10.1038/ismej.2015.125 26296067 PMC4737935

[B3] Burmølle M. Hansen L. H. Sørensen S. J. (2005). Use of a whole-cell biosensor and flow cytometry to detect AHL production by an indigenous soil community during decomposition of litter. *Microb. Ecol.* 50 221–229. 10.1007/s00248-004-0113-8 16195831

[B4] Danovaro R. Dell’Anno A. Corinaldesi C. Magagnini M. Noble R. Tamburini C. (2008). Major viral impact on the functioning of benthic deep-sea ecosystems. *Nature* 454 1084–1087. 10.1038/nature07268 18756250

[B5] Dong Y. H. Gusti A. R. Zhang Q. Xu J. L. Zhang L. H. (2002). Identification of quorum-quenching N-acyl homoserine lactonases from *Bacillus* species. *Appl. Environ. Microbiol.* 68 1754–1759. 10.1128/aem.68.4.1754-1759.2002 11916693 PMC123891

[B6] Dou C. Xiong J. Gu Y. Yin K. Wang J. Hu Y. (2018). Structural and functional insights into the regulation of the lysis–lysogeny decision in viral communities. *Nat. Microbiol.* 3 1285–1294. 10.1038/s41564-018-0259-7 30323253

[B7] Erdönmez D. Rad A. Y. Aksöz N. (2017). Quorum sensing molecules production by nosocomial and soil isolates *Acinetobacter baumannii*. *Arch. Microbiol.* 199 1325–1334. 10.1007/s00203-017-1408-8 28688010

[B8] Erez Z. Steinberger-Levy I. Shamir M. Doron S. Stokar-Avihail A. Peleg Y. (2017). Communication between viruses guides lysis–lysogeny decisions. *Nature* 541 488–493. 10.1038/nature21049 28099413 PMC5378303

[B9] Fuqua C. Greenberg E. P. (2002). Listening in on bacteria: acyl-homoserine lactone signaling. *Nat. Rev. Mol. Cell Biol.* 3 685–696. 12209128 10.1038/nrm907

[B10] Gallego del Sol F. Penadés J. R. Marina A. (2019). Deciphering the molecular mechanism underpinning phage arbitrium communication systems. *Mol. Cell* 74 59–72. 30745087 10.1016/j.molcel.2019.01.025 PMC6458997

[B11] Ghosh D. Roy K. Williamson K. E. Srinivasiah S. Wommack K. E. Radosevich M. (2009). Acyl-homoserine lactones can induce virus production in lysogenic bacteria: an alternative paradigm for prophage induction. *Appl. Environ. Microbiol.* 75 7142–7152. 10.1128/aem.00950-09 19783745 PMC2786502

[B12] Hong K. W. Koh C. L. Sam C. K. Yin W. F. Chan K. G. (2012). Quorum quenching revisited—from signal decays to signalling confusion. *Sensors* 12 4661–4696. 10.3390/s120404661 22666051 PMC3355433

[B13] Howard-Varona C. Hargreaves K. R. Abedon S. T. Sullivan M. B. (2017). Lysogeny in nature: mechanisms, impact and ecology of temperate phages. *ISME J.* 11 1511–1520. 10.1038/ismej.2017.16 28291233 PMC5520141

[B14] Huang J. J. Han J. I. Zhang L. H. Leadbetter J. R. (2003). Utilization of acyl-homoserine lactone quorum signals for growth by a soil pseudomonad and *Pseudomonas aeruginosa* PAO1. *Appl. Environ. Microbiol.* 69 5941–5949. 10.1128/aem.69.10.5941-5949.2003 14532048 PMC201243

[B15] Huang Y. Zeng Y. Yu Z. Zhang J. (2013). Distribution and diversity of acyl homoserine lactone producing bacteria from four different soils. *Curr. Microbiol.* 66 10–15. 10.1007/s00284-012-0234-0 23007525

[B16] Hurwitz B. L. U’Ren J. M. (2016). Viral metabolic reprogramming in marine ecosystems. *Curr. Opin. Microbiol.* 31 161–168. 10.1016/j.mib.2016.04.002 27088500

[B17] Jemielita M. Wingreen N. S. Bassler B. L. (2018). Quorum sensing controls *Vibrio cholerae* multicellular aggregate formation. *eLife* 7:e42057. 10.7554/eLife.42057 PMC6351105 30582742

[B18] Kim M. K. Ingremeau F. Zhao A. Bassler B. L. Stone H. A. (2016). Local and global consequences of flow on bacterial quorum sensing. *Nat. Microbiol.* 1 1–5. 10.1038/nmicrobiol.2015.5 PMC5010089 27571752

[B19] Knowles B. Bailey B. Boling L. Breitbart M. Cobián-Güemes A. Del Campo J. (2017). Variability and host density independence in inductions-based estimates of environmental lysogeny. *Nat. Microbiol.* 2:17064. 10.1038/nmicrobiol.2017.64 28452987

[B20] Knowles B. Silveira C. B. Bailey B. A. Barott K. Cantu V. A. Cobián-Güemes A. G. (2016). Lytic to temperate switching of viral communities. *Nature* 531 466–470. 26982729 10.1038/nature17193

[B21] Kozich J. J. Westcott S. L. Baxter N. T. Highlander S. K. Schloss P. D. (2013). Development of a dual-index sequencing strategy and curation pipeline for analyzing amplicon sequence data on the MiSeq Illumina sequencing platform. *Appl. Environ. Microbiol.* 79 5112–5120. 10.1128/aem.01043-13 23793624 PMC3753973

[B22] Kronheim S. Daniel-Ivad M. Duan Z. Hwang S. Wong A. I. Mantel I. (2018). A chemical defence against phage infection. *Nature* 564 283–286. 10.1038/s41586-018-0767-x 30518855

[B23] Kusada H. Zhang Y. Tamaki H. Kimura N. Kamagata Y. (2019). Novel N-acyl homoserine lactone-degrading bacteria isolated from penicillin-contaminated environments and their quorum-quenching activities. *Front. Microbiol.* 10:455 10.3389/fmicb.2019.00455 PMC6426785 30923518

[B24] Kuzyakov Y. Mason-Jones K. (2018). Viruses in soil: nano-scale undead drivers of microbial life, biogeochemical turnover and ecosystem functions. *Soil Biol. Biochem.* 127 305–317. 10.1016/j.soilbio.2018.09.032

[B25] Laganenka L. Sander T. Lagonenko A. Chen Y. Link H. Sourjik V. (2019). Quorum sensing and metabolic state of the host control lysogeny-lysis switch of bacteriophage T1. *mBio* 10:e01884-19. 10.1128/mBio.01884-19 PMC6737242 31506310

[B26] Li J. Azam F. Zhang S. (2016). Outer membrane vesicles containing signalling molecules and active hydrolytic enzymes released by a coral pathogen *Vibrio shilonii* AK1. *Environ. Microbiol.* 18 3850–3866. 10.1111/1462-2920.13344 27102379

[B27] Liang X. Radosevich M. (2019). Commentary: a host-produced quorum-sensing autoinducer controls a phage lysis-lysogeny decision. *Front. Microbiol.* 10:1201 10.3389/fmicb.2019.01171 PMC6558226 31231325

[B28] Liang X. Zhang Y. Wommack K. E. Wilhelm S. W. DeBruyn J. M. Sherfy A. C. (2020). Lysogenic reproductive strategies of viral communities vary with soil depth and are correlated with bacterial diversity. *Soil Biol. Biochem.* 6:107767 10.1016/j.soilbio.2020.107767

[B29] Liang X. Wagner R. E. Li B. Zhang N. Radosevich M. (2019a). Prophage induction mediated by quorum sensing signals alters soil bacterial community structure. *bioRxiv* [Preprint]. 10.1101/805069

[B30] Liang X. Wagner R. E. Zhuang J. DeBruyn J. M. Wilhelm S. W. Liu F. (2019b). Viral abundance and diversity vary with depth in a southeastern United States agricultural ultisol. *Soil Biol. Biochem.* 137:107546 10.1016/j.soilbio.2019.107546

[B31] Liang X. Zhuang J. Löffler F. E. Zhang Y. DeBruyn J. M. Wilhelm S. W. (2019c). Viral and bacterial community responses to stimulated Fe (III)-bioreduction during simulated subsurface bioremediation. *Environ. Microbiol.* 21 2043–2055. 10.1111/1462-2920.14566 30773777

[B32] Liao L. Schaefer A. L. Coutinho B. G. Brown P. J. Greenberg E. P. (2018). An aryl-homoserine lactone quorum-sensing signal produced by a dimorphic prosthecate bacterium. *Proc. Natl. Acad. Sci. U.S.A.* 115 7587–7592. 10.1073/pnas.1808351115 29967162 PMC6055194

[B33] Ling J. Zhu R. Laborda P. Jiang T. Jia Y. Zhao Y. (2019). LbDSF, the *Lysobacter brunescens* quorum sensing system diffusible signaling factor, regulates anti-Xanthomonas XSAC biosynthesis, colony morphology, and surface motility. *Front. Microbiol.* 10:1230 10.3389/fmicb.2019.01230 PMC6591275 31275253

[B34] Loh B. Kuhn A. Leptihn S. (2019). The fascinating biology behind phage display: filamentous phage assembly. *Mol. Microbiol.* 111 1132–1138. 10.1111/mmi.14187 30556628

[B35] Loudon A. H. Woodhams D. C. Parfrey L. W. Archer H. Knight R. McKenzie V. (2014). Microbial community dynamics and effect of environmental microbial reservoirs on red-backed salamanders (*Plethodon cinereus*). *ISME J.* 8 830–840. 10.1038/ismej.2013.200 24335825 PMC3960541

[B36] Love M. I. Huber W. Anders S. (2014). Moderated estimation of fold change and dispersion for RNA-seq data with DESeq2. *Genome Biol.* 15:550. 10.1186/s13059-014-0550-8 PMC4302049 25516281

[B37] Maslov S. Sneppen K. (2017). Population cycles and species diversity in dynamic Kill-the-Winner model of microbial ecosystems. *Sci. Rep.* 7:39642. 10.1038/srep39642 PMC5209715 28051127

[B38] McCready A. R. Paczkowski J. E. Henke B. R. Bassler B. L. (2019). Structural determinants driving homoserine lactone ligand selection in the *Pseudomonas aeruginosa* LasR quorum-sensing receptor. *Proc. Natl. Acad. Sci. U.S.A.* 116 245–254. 10.1073/pnas.1817239116 30559209 PMC6320529

[B39] McMurdie P. J. Holmes S. (2014). Waste not, want not: why rarefying microbiome data is inadmissible. *PLoS Comput. Biol.* 10:e1003531 10.1371/journal.pcbi.1003531 PMC3974642 24699258

[B40] Miller M. B. Bassler B. L. (2001). Quorum sensing in bacteria. *Annu. Rev. Microbiol.* 55 165–199. 11544353 10.1146/annurev.micro.55.1.165

[B41] Oh J. H. Alexander L. M. Pan M. Schueler K. L. Keller M. P. Attie A. D. (2019). Dietary fructose and microbiota-derived short-chain fatty acids promote bacteriophage production in the gut symbiont *Lactobacillus reuteri*. *Cell Host Microbe* 25 273–284. 30658906 10.1016/j.chom.2018.11.016

[B42] Oksanen F. G. B. Friendly M. Kindt R. Legendre P. McGlinn D. Minchin P. R. (2016). *vegan: Community Ecology Package. R Package Version 2.2-0.*

[B43] Payet J. P. Suttle C. A. (2013). To kill or not to kill: the balance between lytic and lysogenic viral infection is driven by trophic status. *Limnol. Oceanogr.* 58 465–474. 10.4319/lo.2013.58.2.0465

[B44] Polkade A. V. Mantri S. S. Patwekar U. J. Jangid K. (2016). Quorum sensing: an under-explored phenomenon in the phylum *Actinobacteria*. *Front. Microbiol.* 7:131 10.3389/fmicb.2016.00131 PMC4748050 26904007

[B45] Quast C. Pruesse E. Yilmaz P. Gerken J. Schweer T. Yarza P. (2013). The SILVA ribosomal RNA gene database project: improved data processing and web-based tools. *Nucleic Acids Res.* 41 D590–D596. 23193283 10.1093/nar/gks1219 PMC3531112

[B46] Rakonjac J. Feng J. N. Model P. (1999). Filamentous phage are released from the bacterial membrane by a two-step mechanism involving a short C-terminal fragment of pIII. *J. Mol. Biol.* 289 1253–1265. 10.1006/jmbi.1999.2851 10373366

[B47] Reyes A. Haynes M. Hanson N. Angly F. E. Heath A. C. Rohwer F. (2010). Viruses in the fecal microbiota of monozygotic twins and their mothers. *Nature* 466 334–338. 10.1038/nature09199 20631792 PMC2919852

[B48] Rossmann F. S. Racek T. Wobser D. Puchalka J. Rabener E. M. Reiger M. (2015). Phage-mediated dispersal of biofilm and distribution of bacterial virulence genes is induced by quorum sensing. *PLoS Pathog.* 11:e1004653 10.1371/journal.ppat.1004653 PMC4338201 25706310

[B49] Silpe J. E. Bassler B. L. (2019a). A host-produced quorum-sensing autoinducer controls a phage lysis-lysogeny decision. *Cell* 176 268–280. 30554875 10.1016/j.cell.2018.10.059 PMC6329655

[B50] Silpe J. E. Bassler B. L. (2019b). Phage-encoded LuxR-type receptors responsive to host-produced bacterial quorum-sensing autoinducers. *mBio* 10:e00638-19. 10.1128/mBio.00638-19 PMC6456758 30967469

[B51] Stokar-Avihail A. Tal N. Erez Z. Lopatina A. Sorek R. (2019). Widespread utilization of peptide communication in phages infecting soil and pathogenic bacteria. *Cell Host Microbe* 25 746–755. 31071296 10.1016/j.chom.2019.03.017 PMC6986904

[B52] Sullivan M. B. Weitz J. S. Wilhelm S. (2017). Viral ecology comes of age. *Environ. Microbiol. Rep.* 9 33–35. 10.1111/1758-2229.12504 27888577

[B53] Thingstad T. F. Lignell R. (1997). Theoretical models for the control of bacterial growth rate, abundance, diversity and carbon demand. *Aquat. Microb. Ecol.* 13 19–27. 10.3354/ame013019

[B54] Tian J. He N. Kong W. Deng Y. Feng K. Green S. M. (2018). Deforestation decreases spatial turnover and alters the network interactions in soil bacterial communities. *Soil Biol. Biochem.* 123 80–86. 10.1016/j.soilbio.2018.05.007

[B55] Toyofuku M. Morinaga K. Hashimoto Y. Uhl J. Shimamura H. Inaba H. (2017). Membrane vesicle-mediated bacterial communication. *ISME J.* 11 1504–1509. 10.1038/ismej.2017.13 28282039 PMC5437348

[B56] Vinoj G. Vaseeharan B. Thomas S. Spiers A. J. Shanthi S. (2014). Quorum-quenching activity of the AHL-lactonase from *Bacillus licheniformis* DAHB1 inhibits *Vibrio* biofilm formation in vitro and reduces shrimp intestinal colonisation and mortality. *Mar. Biotechnol.* 16 707–715. 10.1007/s10126-014-9585-9 25060960

[B57] Wang Q. Guan Z. Pei K. Wang J. Liu Z. Yin P. (2018). Structural basis of the arbitrium peptide–AimR communication system in the phage lysis–lysogeny decision. *Nat. Microbiol.* 3 1266–1273. 10.1038/s41564-018-0239-y 30224798

[B58] Weitz J. S. Dushoff J. (2008). Alternative stable states in host–phage dynamics. *Theor. Ecol.* 1 13–19. 10.1007/s12080-007-0001-1

[B59] Wellington S. Greenberg E. P. (2019). Quorum sensing signal selectivity and the potential for interspecies cross talk. *mBio* 10:e00146-19. 10.1128/mBio.00146-19 PMC6401477 30837333

[B60] Wickham H. (2016). *ggplot2: Elegant Graphics for Data Analysis.* Berlin: Springer.

[B61] Williamson K. E. Fuhrmann J. J. Wommack K. E. Radosevich M. (2017). Viruses in soil ecosystems: an unknown quantity within an unexplored territory. *Annu. Rev. Virol.* 4 201–219. 28961409 10.1146/annurev-virology-101416-041639

[B62] Williamson K. E. Radosevich M. Smith D. W. Wommack K. E. (2007). Incidence of lysogeny within temperate and extreme soil environments. *Environ. Microbiol.* 9 2563–2574. 10.1111/j.1462-2920.2007.01374.x 17803780

[B63] Williamson K. E. Radosevich M. Wommack K. E. (2005). Abundance and diversity of viruses in six Delaware soils. *Appl. Environ. Microbiol.* 71 3119–3125. 10.1128/aem.71.6.3119-3125.2005 15933010 PMC1151856

[B64] Xue H. Xu Y. Boucher Y. Polz M. F. (2012). High frequency of a novel filamentous phage, VCYϕ, within an environmental *Vibrio cholerae* population. *Appl. Environ. Microbiol.* 78 28–33. 10.1128/aem.06297-11 22020507 PMC3255608

[B65] Yoon J. H. Lee J. K. Jung S. Y. Kim J. A. Kim H. K. Oh T. K. (2006). *Nocardioides kongjuensis* sp. nov., an N-acylhomoserine lactone-degrading bacterium. *Int. J. Syst. Evol. Microbiol.* 56 1783–1787. 10.1099/ijs.0.64120-0 16902008

