# Supplementary material for: Quorum Sensing Signals Alter in vitro Soil Virus Abundance and Bacterial Community Composition
Source: Front Microbiol. 2020 Jun 10;11:1287. doi: 10.3389/fmicb.2020.01287 (PMC7298970; doi:10.3389/fmicb.2020.01287)
Supplement: Supplementary file 1 [file Data_Sheet_1.docx]

**Supplementary information**

**Quorum sensing signals alter in vitro soil virus abundance and bacterial community composition**

Xiaolong Liang^a*^, Regan E. Wagner^a^, Bingxue Li^b^, Ning Zhang^c^, and Mark Radosevich^a*^

*^a^ Department of Biosystems Engineering and Soil Science, The University of Tennessee, Knoxville, TN 37996, United States*

*^b^ College of Land and Environment, Shenyang Agricultural University, Shenyang, Liaoning, China*

*^c^ College of Biotechnology, Shenyang Agricultural University, Shenyang, Liaoning, China*

*Corresponding author

**Figure S1.** Experimental procedure.


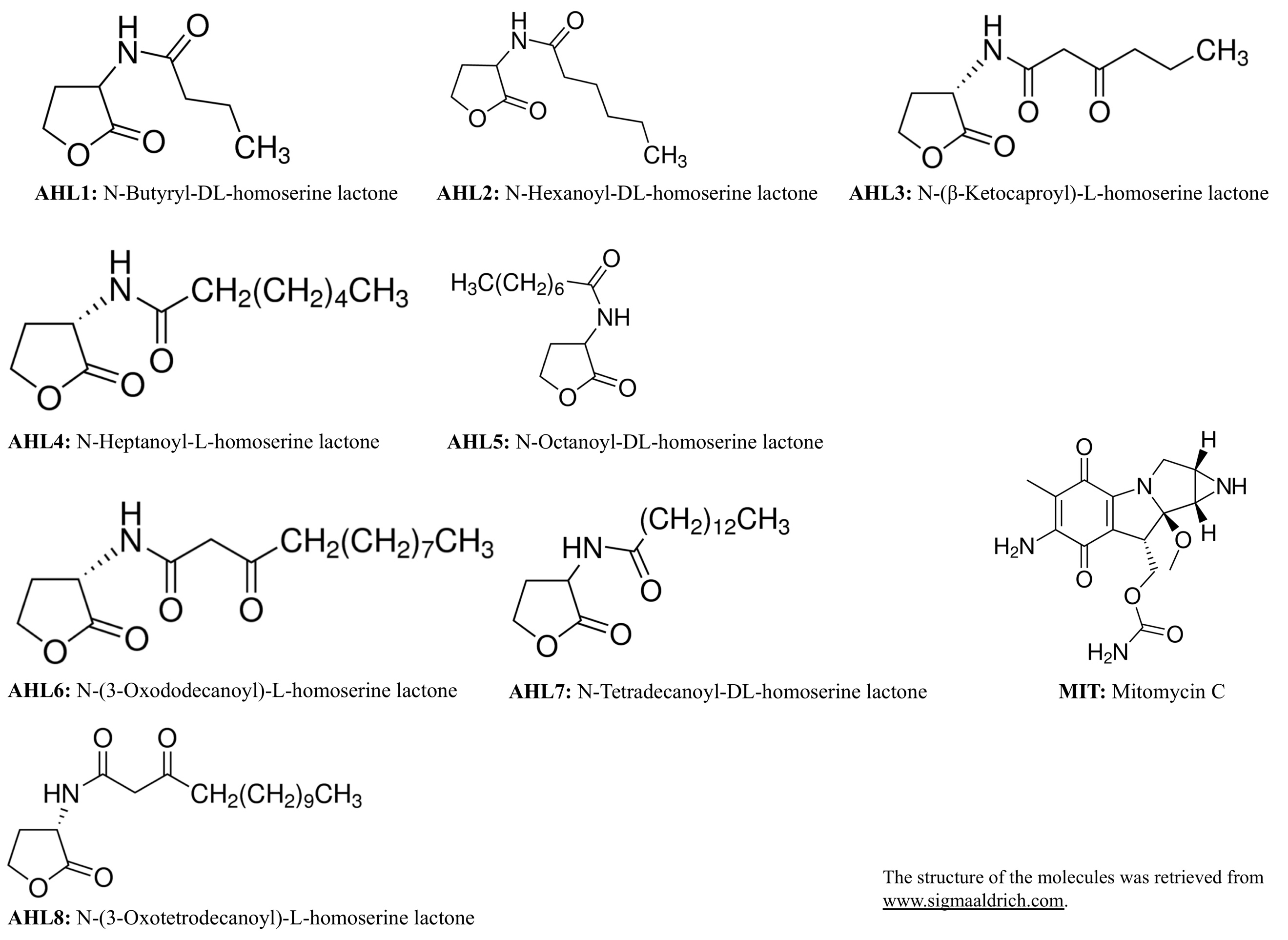


**Figure S2.** The molecular structure of quorum-sensing N-Acyl homoserine lactones (AHL1–8) and mitomycin C (MIT).


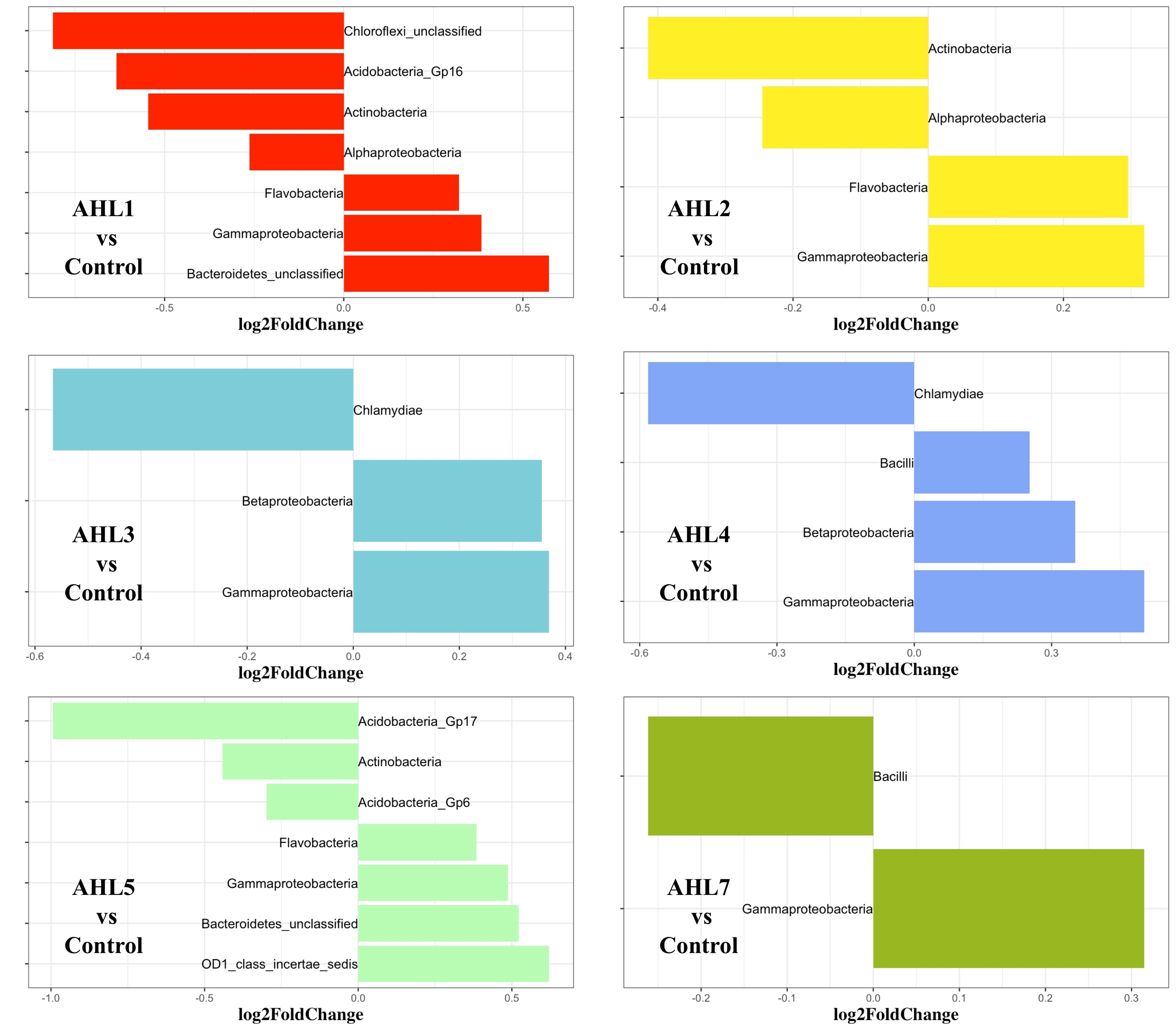


**Fig. S3** Class-level differences of the bacterial community composition between each induction assay of N-(Butyryl, Hexanoyl, β-Ketocaproyl, Heptanoyl, Octanoyl, 3-Oxododecanoyl, Tetradecanoyl, and 3-Oxotetradecanoyl) homoserine lactones (shown as AHL1–8, respectively) and the control cell suspensions. Only statistically significant differences (*P* < 0.01) are shown. The direction of bars represents decrease (left) or increase (right) in the relative abundance of the specific bacterial taxonomic groups after the induction assays.


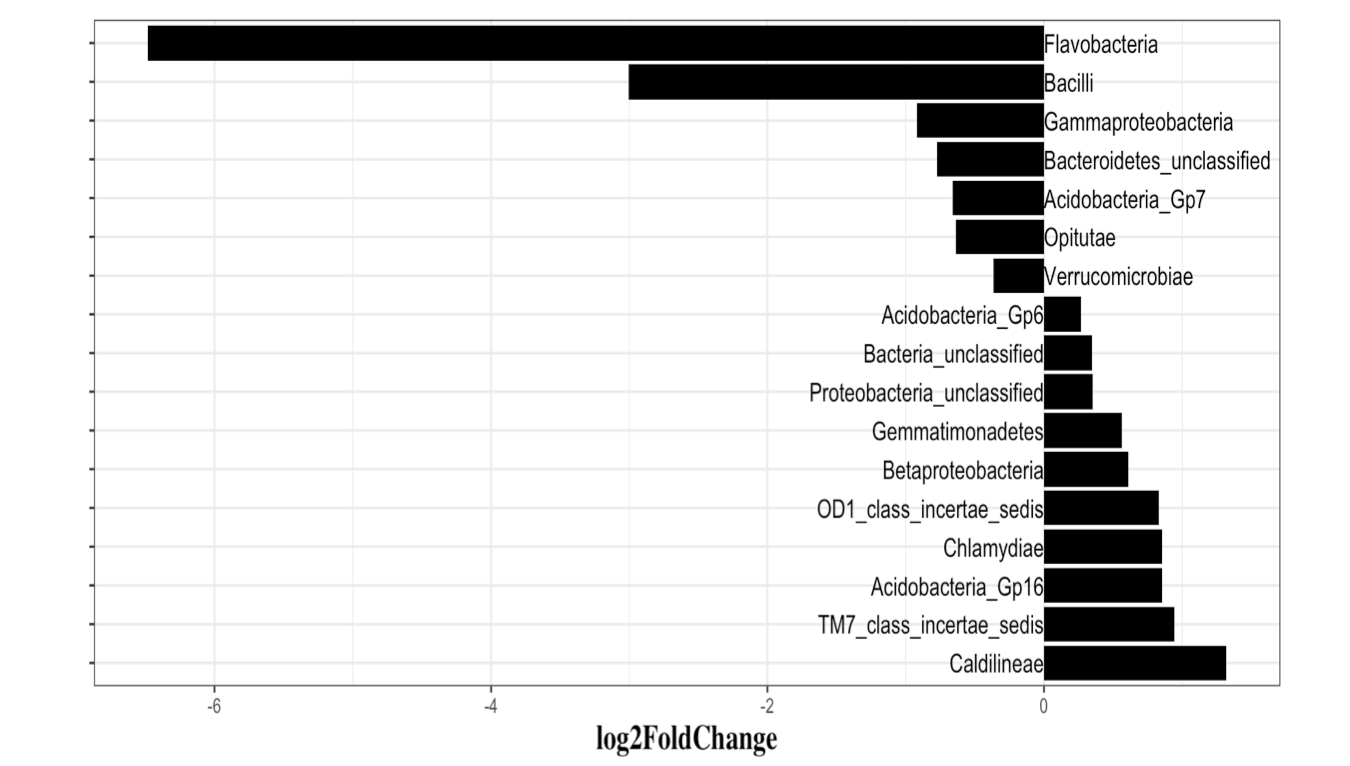


**Fig. S4** Systematic differences of the bacterial taxonomic composition between induction assay of mitomycin C and the control samples at Class levels. Only statistically significant differences (*P* < 0.01) are shown. The direction of bars represents decreases (left) or increases (right) in relative abundance of the specific bacterial taxonomic groups after the induction assays.
